# The Situation of Cervical Cancers in the Context of Female Genital Cancer Clustering and Burden of Disease in Arad County, Romania

**DOI:** 10.3390/jcm8010096

**Published:** 2019-01-15

**Authors:** Ana-Liana Tataru, Gheorghe Furau, Jompan Afilon, Cringu Ionescu, Mihai Dimitriu, Ovidiu Gabriel Bratu, Delia Mirela Tit, Simona Bungau, Cristian Furau

**Affiliations:** 1 Department of Obstetrics and Gynecology, Arad County Clinical Hospital, 310023 Arad, Romania; ana.liana.tataru@gmail.com (A.-L.T.); gfurau@yahoo.com (G.F.); cristianfurau@gmail.com (C.F.); 2 Department of Life Sciences, “Vasile Goldis” Western University of Arad, 310045 Arad, Romania; 3 Department of General Medicine, “Vasile Goldis” Western University of Arad, 310045 Arad, Romania; 4 Department of Doctoral School, “Vasile Goldis” Western University of Arad, 310045 Arad, Romania; ajompan@uvvg.ro; 5 Clinical Department 13, “Carol Davila” University of Medicine and Pharmacy, 050474 Bucharest, Romania; antoniuginec@yahoo.com (C.I.); drmihaidimitriu@yahoo.com (M.D.); 6 Clinical Department 3, “Carol Davila” University of Medicine and Pharmacy, 050474 Bucharest, Romania; ovi78doc@yahoo.com; 7 Department of Pharmacy, University of Oradea, 410028 Oradea, Romania; mirela_tit@yahoo.com

**Keywords:** disability-adjusted life years, women, genital cancer, cervical pathology, quality of life

## Abstract

Romania has the highest incidence of cervical cancer morbidity and mortality in Europe. This study identifies the major clusters for genital cancers, observes the features of genital and cervical cancer, and determines the extent to which cancer is a contributor to total Disability-Adjusted Life Year (DALY). Spatial analysis used Besag and Newell’s method for genital cancer distribution, prevalence considered Arad County patients records (2008–2017), and DALY was determined according to WHO methodology and GLOBOCAN 2013 data. Diagnosis was established by histopathological examination of diagnostic biopsies or tissues obtained by surgical procedures, followed by clinical staging. 1695 women were recorded with genital cancer. Of these, 14.9% of lesions were in situ (*n* = 252) and 74.20% of cases were recorded in stage III or IV (*n* = 1258) (*p* < 0.0001). Over 90% of cervical cancers were squamous cell carcinomas (*n* = 728), 33.76% of endometrial cancers were adenocarcinomas in situ (*n* = 131), 32.42% of ovarian cancers were serous adenocarcinomas (*n* = 131), and 70.58% of vulvar cancers were squamous cell carcinomas (*n* = 48) (*p* < 0.0001). DALY/1000 was 67.2 for genital cancers and 33 for cervical cancers. From the point of view of Romanian women, cervical cancer remains one of the major problems that need to be dealt with and access to optimal treatment proves to be extremely limited.

## 1. Introduction

Cervical cancer is one of the most preventable cancers. In 2012, GLOBOCAN estimated 58,300 new cases of cervical cancer in Europe, with a standardized incidence rate of 13.4 per 100,000 and 24,400 deaths in Europe, with a standardized mortality rate of 4.9 [1]. Age-standardized rate (ASR) for cervical cancer in Europe is 11.4 per 100,000 women/year, compared to 28.6 per 100.000 for Romania. For Arad Region (a western area of Romania), there is a cancer database from 1960 which was adapted after 2007 requirements [2]. In the last ten years, cervical cancer incidence in Arad exceeds national values, being 3 times greater than the average of Europe. Romania ranks first in the European Union countries in terms of cervical cancer mortality, while cervical cancer survivors are dealing each day with sequelae related to their disease [3].

Vaginal and vulvar cancers are rare and characteristic for elderly women. The most common histopathological type is squamous carcinoma. Vulvar cancers are usually diagnosed at an early stage with a relatively good prognosis and a survival rate over 5 years for nearly 80% of cases [4]. The cancer found in the vagina is more often metastatic (cervical, endometrial, ovarian, colorectal) rather than non-metastatic. Endometrial cancer is the most common female neoplastic pathology in the US, with main risk factors being obesity and old age. We also observed an increase of this type of cervical cancer as obesity becomes more frequent in both our country (Romania) and county (Arad). Because of vaginal bleeding, patients are consulting healthcare professionals in early stages, with a good survival rate. Uterine sarcoma and carcinosarcoma tend to rapidly increase, lymphatic and haematogenic dissemination are premature, and prognosis is reserved. Ovarian cancer is responsible for a significant number of deaths, considering the lack of edifying symptoms for early diagnosis and the absence of specific screening tests. Trophoblastic gestational neoplasia is a subcategory of gestational trophoblastic disease that evolves in malignant form. Prognosis is favorable and usually patients heal despite distant metastases.

Cervical cancer, especially squamous carcinoma, develops most frequently at the level of squamo-columnar junction (SCJ) from a pre-existing lesion, generally occurring consecutively with HPV infection [4]. It is considered that, during embryogenesis, the upward migration of the stratified squamous epithelium from the urogenital sinus and the vaginal plateau replaces the Mullerian epithelium. Normally, this process ends near the external cervical orifice, forming a squamo-columnar junction. For a small number of women, this migration can be incomplete and leads to a localization of the squamo-columnar junction in the upper portion of the vagina. The cylindrical epithelium is known as “glandular” epithelium. This is due to the profound invagination of the columnar epithelium, which determines a histopathological aspect similar to that of the glandular tissue. The localization of the squamo-columnar junction varies with age and hormonal status [4]. This squamo-columnar junction is, in most of the cases, accessible for clinical investigation, allowing efficient screening programs (PAP smear—Papanicolaou test, liquid PAP, HPV typing). Because worldwide cervical cancer affects 490,000 new women each year, many countries have implemented such programs and results are reflected in their lower morbidity and mortality (only about 1550 Canadian women were diagnosed with cervical cancer in 2017, while every year 10,000 Japanese women are newly diagnosed with cervical cancer) [5,6].

Almost all cervical malignancies, squamous or cylindrical, develops in the transformation area, adjacent to the squamo-columnar junction. The reserve cervical cells and immature metaplastic cells appear particularly vulnerable to the oncogenic effects of HPV and carcinogens [7]. 

The human Papilloma virus is the etiological agent of cervical cancer, but it also has a decisive role in the occurrence of vaginal, vulvar, anal, and pharyngeal cancers. The onset of sexual life exposes the woman to the risk of acquiring one or more HPV strains. The risk of infection is maintained throughout life, but the prevalence is at maximum in women under 25 years of age. Approximately 20% of HPV-infected women develop preprocessor lesions of type CIN (cervical intraepithelial neoplasia). Most women with sustained injuries will remove the virus later and this purge will result in the regression of the sustained lesions. If the virus is not purged, the persistence of high-risk HPV oncogenic genotype infections (16, 18, 31, 33, 35, 45, 58) will lead to the development of a CIN 3 injury, which (after a variable period of 8–13 years) will result in an invasive carcinoma. One of the most important elements of carcinogenesis in the cervix is a persistent infection (over 6–12 months) with an HPV oncogene [7]. Other important risk factors for cervical cancer are smoking, contraceptive pills, HIV, induced immunodepression or medication post-transplant organ, early age of first sexual contact, and multiple partners [7].

The classification of cervical lesions follows the BETHESDA system [8] and comprises the following lesions:

- Squamous Cell: atypical squamous cells of undetermined significance (ASC-US) and atypical squamous cells—cannot exclude high-grade squamous intraepithelial lesion (HSIL) or atypical squamous cells high-grade (ASC-H); low grade squamous intraepithelial lesion (LSIL or LGSIL) encompassing: HPV/mild dysplasia/CIN 1; HSIL encompassing: moderate and severe dysplasia, CIS, CIN 2, and CIN 3; squamous cell carcinoma.
- Glandular Cell: atypical endocervical, endometrial, glandular cells; endocervical adenocarcinoma in situ; adenocarcinoma.

L-SIL dysplasia is associated with high-risk HPV oncogenic genotype infections. From the histopathological point of view, they correspond to the type 1 lesions, but there are rare situations when CIN 2 and CIN 3 lesions can be detected. H-SIL dysplasia are associated with a risk of moderate dysplasia (CIN 2), but also of preinvasive lesions (CIN 3) in 70% of cases, or even invasive in 1–2% of cases [7]. Nowadays, genetic testing and biomarkers are also available, but they are still expensive and not fully covered by the public health insurance, and therefore only a few women can benefit and receive proper therapy in early stage.

### Magnitude and Peculiarities of the Problem

Arad County is characterized by its annual incidence for all cancers, with 413.6 cases per 100,000 inhabitants in 2016, compared to the average in Romania of 298.8 cases per 100,000 [9]. The natural history of cervical cancer in Arad County, as documented with the records of the oncology section, started in 1957; it records 2333 cases between 1957 and 2017 with uneven spatial distribution, forming clusters, established by spatial analysis using Besag and Newell’s method [10] applied to the whole of Arad County. The principle of the method assumes, as null hypothesis, a normal Poisson distribution for cancer cases. Where the distribution of the cases is increased, the working hypothesis occurs, which contradicts the null hypothesis, and the population in which the occurrence of cancers defies the normal distribution represents a cluster [11], depending on the cut-off value k determined by the researchers [12]. Because it is stated that only 10% of the statistical clusters are real clusters of disease, these situations should be investigated for the identification of risk factors for cancers. The histology of cervical cancers proves not only a high incidence, but there appear to be an unusual number of rare type cervical cancers documented, such as some variants of endometrial neoplasia (clear-cell tumors), undifferentiated carcinomas (which is extremely rare and aggressive), Ewing’s cervix sarcoma (documented in only 18 cases in the Anglo-Saxon literature), cervix verrucous carcinoma, epidermoid carcinoma, or cervical rhabdomyosarcoma (very rare in the adult), all of them being present in the cervical pathology of Arad County.

The burden of the disease is very important both for patient and society. For the patient, we can refer to DALY as an efficient measuring tool, but for the society, the assessment of the effect is more difficult as it involves the direct cost, the family, the work environment, the social activities, and the psychological problems. One DALY can be thought of as one lost year of “healthy” life. The sum of these DALYs across the population or the burden of disease can be thought of as a measurement of the gap between current health status and an ideal health situation, where the entire population lives to an advanced age, free of disease and disability. DALYs for a disease or health condition are calculated as the sum of the Years of Life Lost (YLL) due to premature mortality in the population and the Years Lost due to Disability (YLD) for people living with the health condition or its consequences [13]. 

Quality adjusted life years (QALY) measures years lived in perfect health gained and is the arithmetic product of life expectancy combined with a measure of the quality of life-years remaining. QALYs can lack sensitivity and may be difficult to apply to chronic disease and preventative treatment [14]. We preferred to use DALY because this type of analysis provides accurate data. The other system requires comparisons with other studies, and such QALY studies have not been done in Romania.

This is a retrospective study which has been carried out in Arad County, on the histopathological analysis of samples obtained through diagnostic biopsies or surgery for genital pathology, in order to determine the frequency and characteristics of the female subpopulation most affected by genital cancer during 2008–2017, and to determine the extent to which this cancer is a contributor to total DALYs.

## 2. Experimental Section

### 2.1. Study Design

A total of 1695 cases of feminine genital cancer (registered between 2008 and 2017 in the only oncology service of the Arad County Emergency Hospital) have been analyzed, from the point of view of death rates, stage of cancer, histological characterization, as well as PYLL (Years of potential life lost), YLL, and YLD during this period. Arad County Cancer Register Database was the starting point for the selection of the cases, all cases registered being processed. Inclusion criteria were: female, resident in Arad County for at least 10 years prior to cancer diagnosis, anatomopathological requirements for in situ or genital cancer lesions. The selected patients had the clinical and histopathological diagnosis of primary genital cancer in the obstetrics-gynecology department of Arad Emergency Hospital (metastatic genital tumors were not included). Once the criteria of addressability to specialized gynecology services (specific symptomatology, medical diagnosis, anatomopathological diagnosis, specific therapy through gynecological surgery, cooperation with the oncology service) were met, the selected cases were enrolled in the obstetrics-gynecology department of Arad Emergency Hospital, who fulfilled the diagnostic criteria admission for genital cancer, for all cases. 

The research was conducted in accordance with the WMA Declaration of Ethical Helsinki—Medical Research Involving Human Principles for Subjects, and received the ethical approval from the Ethical Council of the Emergency Clinical County Hospital of Arad (No. 13/10.01.2017). Considering that this is a retrospective study, signed informed consent was not required. Furthermore, in the period we studied, all patients admitted in obstetrics-gynecology department were questioned about processing medical data for national health statistics, and only cases with patient informed consents could be processed.

### 2.2. Spatial Cancers Distribution 

Spatial cluster analysis was performed using the Besag and Newell’s method applied to region-centered group-level. As a basic statistical test, null hypothesis and work hypothesis were defined. The null hypothesis H0: the cases within the studied area comply with Poisson distribution, assumed as normal. The work hypothesis: there are areas where the number of cases exceeds that predicted by Poisson distribution (considered as normal), which contradicts H0. The spatial area with higher cases densities than the normally expected occurrence is a cluster region. For testing H0, around each region, a circular window is considered, sized to include a specified number of cases k. Population inside the window is compared to the normal Poisson distribution. When the frequency of cases is higher than expected, H0 is rejected, the work hypothesis is accepted, and that specific region is considered a cluster. The t test was used to compare two different averages (means) for statistical significance (*p* = 0.005). For spatial cluster analysis, Arad region was divided into 75 administrative locations as potential clusters, identified by latitude and longitude coordinates. The number of new cases and the population at risk for each region were recorded using Midpoint female population estimation living there more than 10 years. The file was set-up in ClusterSeer software [15] text format, and we established the size of the circular window in successive attempts.

### 2.3. Female Genital Cancers Characterization 

The cancer diagnosis was established by histopathological examination of diagnostic biopsies or tissues taken from surgery procedures followed by clinical staging. The tissues were fixed with formalin and embedded in paraffin. The sections obtained were stained with hematoxylin-eosin and the interpretation was performed on ZEISS Primo Star Optic Microscope (Carl Zeiss, Göttingen, Germany). Later, some of the cases were referred to the tertiary center for immunohistochemistry or other advanced tests. Challenges in this field lie in the heterogeneity in the levels of homologous recombination proteins in different types of tumors, the lack of reliable biomarkers to validate resistance to the inhibitors, development of early molecular diagnostic tools, and the evolution of cancer cell resistance to acquiring additional mutations [16].

The study was focused on genital cancer profiles and age-related relative risk (ARR) for genital cancers. All the cases were analyzed for the quantitative variables. The statistical data were translated into Excel (2016), IBM SPSS Statistic 20, and MedCalc (version 14.8.1) for analysis. Tables and charts have been designed in these programs. We applied the descriptive statistics methods (demographic data, age, residence, diagnostic categories, rates, ratios, percentages, frequencies, mean, median, variance, standard deviation) and analytical methods for cohort observational, longitudinal retrospective, non-randomized study.

### 2.4. DALY 

The Global Burden of Disease GBD 2013 methodology was used. Data sources were: Arad County Cancer Registry, tables for life expectancy 2015 by age categories [17], and those of the Global Health Observatory data repository. YLDs were calculated by multiplying the estimated prevalence by the weight of derived disability. YLLs were calculated by multiplying the estimated deaths for each age category, by life expectancy (LE), and DALY was calculated as sum of YLD and YLS. Target population was the female population from Arad County susceptible to genital cancer. Available population was the female population in the official demographic records, according to “Romanian population by localities on January 1, 2016” [18]. The sample consisted of the cases registered in the oncology department of Arad Emergency Hospital between 2008 and 2017 and treated in the obstetrics-gynecology department.

## 3. Results

### 3.1. Genital Cancer Clusters 

**Figure 1:**
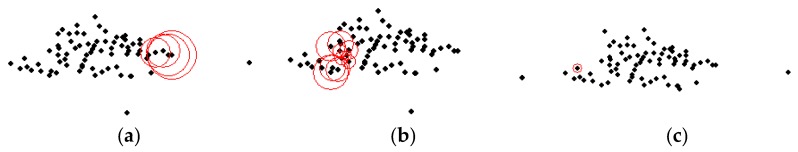
Clusters of cervical cancer. (**a**) Cervical cancer cut-off 75 cases; (**b**) endometrial cancer cut-off 350 cases; (**c**) ovarian cancer cut-off 6 cases.

We identified 12 statistical clusters of cervical cancer (Figure 1a–c) located in the eastern part of the county, three of uterine cancer located in the western half of the county, and one for ovarian cancer also located in the west. 

Cluster distribution results need to be further analyzed to identify potential risk factors and to demonstrate whether they are real disease clusters or just statistical ones.

### 3.2. Descriptive Analysis of Genital Cancers in Women 2008–2017, Arad County

Incidence density rates of genital cancers were characteristic for each type of cancer and age category, with maximum values for different types of cancer—cervical cancer in 50–54 years (89.29), ovarian cancer in 70–74 years (52.2), endometrial cancer in 60–64 years (50.9), vulvar cancer in 75–79 years (16.73), vaginal cancer in 80–84 years (7.62), uterine sarcoma in 55–59 years (2.38)—are presented in Table 1.

ARR for genital cancers was higher for those aged over 50 of the female population compared to those aged under 50 (Table 2).

Early lesion detection is extremely important for a favorable resolution of the disease. Unfortunately, the presentation of the patient to the doctor occurs in late stages of the disease. In situ lesions were 14.86% of all stages, early detection in situ stages being more successful in endometrial cancer, and paradoxically in ovarian cancer. Histopathological types were squamous-cell carcinomas (44.9%), endometrioid adenocarcinomas (12%), and adenocarcinomas (9.67%), as shown in Table 3. 

An extensive clinical and histopathological staging can be seen in Appendix A (Table A1 and Table A2). Death rates exceed half of the cases for all genital cancer types, excepting endometrial ones, as is depicted in Table 4. 

The PYLL, as an indicator of premature mortality, represents the total number of years lost by one woman who died before the age of 78.5 [20], and was calculated for each patient who died before this age. This indicator gives more importance to the causes of death that occurred at younger ages than those occurring at older ages. Deaths occurring in individuals aged 78.5 or older were not included in the calculation. All potential lost years by genital cancers deaths in Arad County during 2008–2017 decade were 22,708 years. Furthermore, 61.33% of them were lost because of cervical cancer (13,927 years), at an average of 31.79 PYLL per death. It has been noticed that cervical and ovarian cancer presented the highest PYLL rates.

### 3.3. DALY for Genital Cancers in Women

YLL consider the age at which deaths occur by giving greater emphasis to deaths at younger age and lower importance to deaths at older age. YLLs are calculated from the number of deaths multiplied by a standard life expectancy at the age at which death occurs. The standard life expectancy used for YLL at each age is the same for deaths in all regions of the world and is the same as that used for the calculation of DALY. 

To estimate YLD for a cause in a particular time period, the number of incident cases in that period is multiplied by the average duration of the disease, and a weight factor that reflects the severity of the disease on a scale from 0 (perfect health) to 1 (dead). The basic formula for YLD is:

YLD = I × DW × L(1)

where I = number of incident cases, DW = disability weight, and L = average duration of the case until remission or death (years).

DALYs for a disease or health condition are calculated as the sum of the YLL due to premature mortality in the population and the YLD for people living with the health condition or its consequences. Values for YLL, YLD, and DALY in women aged 20 and over 85 by genital cancer in Arad County are presented in Table 5. 

**Figure A1:**
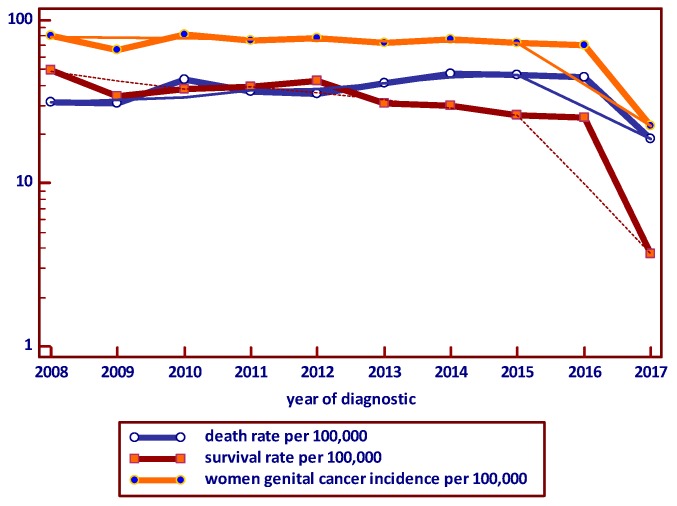
Female genital cancer incidence Arad to 100,000.

Cervical cancer events (*n* = 803) represent half of genital cancer events (*n* = 1695). Age at onset is 60.2 for genital cancers, but it is lower for the cervical ones (56.2). Average duration of the case, until remission or death, is double for cervical cancers (*n* = 4.7 years) compared to the average duration of genital cancers in general (*n* = 2.7 years). YLD by genital cancer is 2103.4 years and by cervical cancer is 350 years. DALY for women aged 20 and over 85 for genital cancer (*n* = 243,921) is 16,397.2 and DALY rate per 1000 is 67.2. Also, DALY per 1000 for cervical cancer represents 49.1% of DALY for genital cancer. Female genital cancer incidence per 100,000, death rate incidence, and survival rate incidence per 100,000 can be seen in Appendix B, Figure A1.

## 4. Discussion

Our work focused on cervical cancer in the context of other female genital cancers in order to determine the frequency and characteristics of the subpopulation most affected by genital cancer in Arad County in the years 2008–2017. The profile of female genital cancers has several characteristics in Arad County, Romania: cases have uneven spatial distribution; histology of neoplastic lesions documents several rare types of cancer (clear cell tumors, verrucous carcinoma of the cervix, rhabdomyosarcoma of uterine cervix); cervical cancer screening programs are not fully implemented, and many cases are referred after diagnosis to specialized regional oncological centers.

Romania has the highest incidence [4] of cervical cancer morbidity and mortality in Europe [2], with an average age of 51.83 years, SD 12.8, which also differs from European data, where the peak of pathology is in the age category 25–35 years, even though some countries show an incidence of bipolar cervical cancer with hooks in patients aged between 45 and 65 years [21,22]. 

The present study showed that the genital cancer incidence and related mortality in Romanian women were significantly higher than those registered in Europe; the relative risks for endometrial cancer over 50 years old compared to under 50 years old exceeded more than 14 times, for ovarian cancer by more than 4 times, and for cervical cancer more than double. In Eurostat statistics, Romania has the highest death rate due to cancer of the cervix, namely 16.4 [23]. Regarding DALYs, further studies are required in five years, and if the disease burden will remain unchanged, health programs must be focused on this problem. Our approach resulted in a series of variables that can be used to estimate region and country-specific DALYs, enabling national estimations of DALYs and international comparisons that support priorities in cancer control. Every percentage change in DALYs is welcomed.

No etiology of tumor disease was sought. High-risk HPV infection was not a criterion for case inclusion, even if these types cause approximately 5% of all cancers worldwide [24]. No genetic tests were available at this stage. We suggest typing of human papillomavirus DNA to be included in screening free analysis, covered by National Insurance House.

For endometrial cancer prevention, perimenopausal women should be evaluated, even if there is no routine screening recommended, except for women with HNPCC (hereditary nonpolyposis colorectal cancer). Ovarian cancer is difficult to detect early, and to date, there is no effective screening regimen. Also, there is no certain method to prevent vaginal cancer, but regular pelvic exams and PAP tests should help in detecting a cancer in its early stages. Although screening for vulvar cancer is not recommended, clinicians should evaluate and perform a biopsy on any suspicious vulvar lesions. Current efforts for prevention are aimed at HPV vaccination [25,26,27]. HPV vaccination seems to be an effective primary prophylaxis, but results are still necessary to confirm this. Many countries managed to introduce it efficiently, but in Romania and Arad County as well, the free HPV vaccination campaign was limited (poor vaccination rate).

There is an enormous problem in demonstrating that the burden of cervical cancer in women can be reduced by developing existing and accessible screening programs and by adopting vaccination methods. This impediment arises because the positive impact of the mentioned interventions can be perceived only after a long (at least 10 years) successful period of these actions, and under conditions in which these control methods must be explained and the population must be adherent to them [28]. Furthermore, the impact on the patient’s family with its psychological challenges and the overall impact on society are difficult to compose and subsequently address. DALYs are a useful aid in establishing county-specific agendas regarding cancer control, even if they normally exclude socio-economic, cultural, and environmental factors with serious impact on the overall burden of diseases and with the ability of people to cope, which is more relevant for women than men [29].

There are regions in Arad County where women are more likely to develop a genital cancer, and there are specific age-categories for every cancer type registered. Histopathological cancer types were dominated by squamous-cell carcinomas, endometrioid adenocarcinomas, and adenocarcinomas. Women had to undergo regular pelvic exams and PAP tests. Applying the national guide remains a request, as it comprises adequate measures such as liquid PAP, HPV typing, and colposcopy. New studies suggest the use of multiple biomarkers in cervical cancer management (importin-β, exportin-5, p16, Mcl1, PDL1, and cFLIP) that are not available in Romania [30]. 

The PAP test is very effective in screening for cervical cancer. Cervical cancer screening was implemented by the Ministry of Health in 2012, and its main objective was to reduce the incidence of invasive forms of cervical cancer and specific mortality through cervical cancer [31]. The implementation of the National Cervical Cancer Screening in Arad County shows a decrease in the cases diagnosed in advanced stages of the disease—217 cases in stage IV of the pathology during 2008–2012, compared to 152 cases in stage IV after screening implementation (2013–2017); also, an increase of the incidence of cancers in situ can be observed, from 37 cases prior to screening, to 158 cases after its implementation. Another very clear aspect is that out of a total of 140,896 women aged between 25 and 64 [32], only an average of 900 women were tested annually through national screening, which accounts for only 0.63% of the eligible population.

The cost of primary prevention of cervical cancer is much lower than for secondary and tertiary prevention. HPV vaccination in Romania can be done with Gardasil 4 (for strains 6, 11, 16, 18), which can be administered according to a 2-dose regimen (0.5 mL at 0 and 6 months), or with a 3-dose regimen (0.5 mL at 0, 2, 6 months), or with Gardasil 9 (for 6, 11, 16, 18, 31, 33, 45, 52, 58 strains), in which the primary vaccination schedule consists of 3 separate doses of 0.5 mL administered at 0, 2, and 6 months. Thus, the costs for vaccinating girls against HPV may vary from 200 euros (for Gardasil 4 for the 2-dose regimen) to 400 euros for the administration of 3 doses of Gardasil 9 [33,34]. The vaccine prices were also different in Romania compared to other countries. In Vietnam, the market price of Cervarix is 35.60 USD per dose and the market price of Gardasil in Vietnam is of 55.80 USD per dose, compared to Romania, where the tetravalent vaccine is 115 USD per dose and the 9-valent vaccine is 150 USD per dose [35]. Early diagnosis of risk factors (HPV oncogenic strains), precancerous lesions, or early stage cancers can be achieved using the PAP test, HPV typing, colposcopy, or biopsy. The cost for these investigations differs between 8 EUR for a PAP test, which is carried out annually, around 66 EUR for HPV typing, 44 EUR for colposcopy, and 250 EUR for biopsy of the cervix during a continuous hospitalization admission. Of all these, the Romanian state settles the PAP test through the National screening program or hospital admission and the cost of hospitalization and cervical biopsy for insured persons only. 

After the diagnostics of cervical cancer, the cost of the treatment varies greatly, depending on the stage. Thus, for a stage IA1 cancer, one can opt for the trachelectomy, for which in Romania, the related hospitalization is on average 650 euro. For Stages IA, IB, and IIA, it is necessary to perform a total hysterectomy, which involves a cost of approximately 3300 Euro for hospitalization, surgical intervention, and pre and post-operative care. If after surgery patients require chemotherapy, to these costs on average 2000 euros per year are added. For patients with localized or metastatic invasion, the cost of chemotherapy is 4000 euros in the first year. In summary, in the first year after cervical cancer diagnosis, the total cost varies between 1000 euros for stage IA1 patients, 3600 euros for cervical cancer, or 5600 euros for associated chemotherapy. For advanced cancer stages, costs reach almost 10,000 euros in the first year. There is a very large difference between early diagnosed cancer costs (younger patients) and late diagnosed (such as older women). It should also be emphasized that the treatment is individualized according to the age of the patients, the associated pathologies and the patient’s ability to undergo surgery or adjuvant treatment. According to the literature, postoperative mortality decreases with neoadjuvant chemotherapy instead of surgical intervention [36,37]. Our results showed that cancer clinic and hospital admissions were the two largest drivers of costs in the first year after diagnosis. Those cost categories capture costs associated with cancer-related treatments, such as chemotherapy and cancer-related surgeries. To these costs are also added those for radiotherapy, palliative care, and psychological care. These costs have been calculated as a result of the hospitalization expense accounts from Arad Clinical County Hospital. In Arad County, there is no possibility of radiotherapy, which is why the patients are being referred to other clinics. In addition to these shortcomings, there is excessive loss for the society of the cancerous women, and especially for their families. The burden of the disease is very important, both for the patient and the society. For the patient we can refer to DALYs as an efficient measuring tool, but for the society, the assessment of the effect is more difficult, as it involves the direct cost, the family, the work environment, social activities, and psychological problems.

Summary measures of population health are crucial inputs to guide health system investments and set priorities at global, regional, national, and subnational levels. DALYs can be considered a summary measure of population health based on estimates of premature mortality and non-fatal health loss, originating from the initial Global Burden of Disease (GBD) study in 1993 [38,39,40,41,42]. Additionally, DALYs, in combination with other summary measures, such as healthy life expectancy (HALE), offer relatively simple, yet powerful metrics, against which progress and challenges in improving disease burden and extending healthy lifespans can be effectively monitored over time [43]. Women’s education regarding their health is crucial; they must participate in screening programs, programs that must ensure the investigation of genetic risk for cancer, living and working conditions, the environment in which they live, and risk behavior. Specialists ought to be more concerned with the public health impact of genital cancers and the authorities should extend the regulations to a much more exhaustive approach to the problem. 

Because Romanian National Health Programs don’t support any costs for Breast Cancer (BRCA)-mutation testing, patients’ BRCA status is unknown. Romanian Professional Guidelines for genital cancers are not referring to gene testing in ovarian cancer, even if it accepted that such tests are useful for searching HBOC (Hereditary Breast and Ovarian Cancer) [44,45]. It is well-known that BRCA-mutated cancer may respond differently to certain cancer treatments, such as a targeted therapy, and some BRCA mutations are hereditary, so the risk of developing cancer might be passed on to family members. Some authors consider that approximately 25% of all ovarian, fallopian tube, and peritoneal cancers are caused by a heritable genetic condition. Of these, about one-quarter (6% of all ovarian, fallopian tube, and peritoneal cancers) are caused by genes other than BRCA1 and BRCA2, including many genes associated with the Fanconi anemia pathway or otherwise involved with homologous recombination [43,46]. 

The epidemiology of female genital cancers in Arad in the last 10 years has not been studied so far. This study showed an uneven distribution of the cancer cases with unknown environmental, behavioral, infectious, or genetic causes, which have to be evaluated in the future. HPV infection, as a cervical cancer determinant, was not an item to be analyzed in the absence of a reliable database and social insurance coverage. On the other hand, the DALYs results are obviously the effects of augmented burden of female genital cancers. The anatomopathological profile decreases, with a few exceptions, within the general characteristics of other studies. Recent cancer therapies are based on major advances in molecular biology, cellular and genomic biology. Biomarkers have multiple roles not only in cervical cancer screening programs, but also in assessing local and peripheral cancer spreading and highlighting genome mutations involved in chemical and radiological resistance, helping to select the appropriate treatment [47]. Administration of drug combinations that provide better clinical outcomes than individual therapeutic agents in patients is such a breakthrough that has been successfully transposed into therapy [48].

Insufficient medical education and the lack of knowledge or interest towards health programs aimed to reduce, prevent, or treat a malignant disease in its early stages (HPV vaccination, free PAP smear screening, mammography) contributes to increasing the burden of disease. There is also sociological fear in cervical cancer testing, as well as in BRCA, or other cancers; cancer testing in general is difficult to accept without preliminary training, and diagnostic tests are considered dangerous [9]. Medical examination is an important sociological issue, and the lack of a well-developed medical screening sociology affects public health objectives.

The future implications of female genital cancers, illustrated by the results of this study, relate to the need to complement the approach of female genital pathology by extending diagnostic and treatment measures to genetic exploration of cancer risks, testing oncogenic risk infections, and fully evaluating patients in social, economic, nutritional, and behavioral aspects.

## 5. Conclusions

Our study identifies the major clusters for genital cancers, observes the features of genital and cervical cancer, and determines the extent to which this cancer is a contributor to total Disability-Adjusted Life Year. We concluded that Romanian women in Arad Region have a considerable genital cancer burden, and the present health programs should be adapted for better action in order to control this disease problem.

Comprehensive measures for cancer prevention and treatment should be implemented to reduce this disease burden. Future studies should be conducted over a long period of time, gathering continuous data to evaluate the economic impact and factors influencing the genital cancer burden in women. 

## Figures and Tables

**Table 1:** Incidence density rate of genital cancer in women per 1000.

Age Category	Cervical Cancer	Ovarian Cancer	Endometrial Cancer	Vulvar Cancer	Vaginal Cancer	Uterine Sarcoma	Placenta Cancer
20–24	1.51 (*n* = 2)	0.75 (*n* = 1)	0.00	0.00	0.00	0.00	0.00
25–29	5.02 (*n* = 9)	1.12 (*n* = 2)	0.56 (*n* = 1)	0.00	0.00	0.00	0.00
30–34	12.84 (*n* = 21)	5.50 (*n* = 9)	1.22 (*n* = 2)	0.00	0.00	0.00	0.00
35–39	25.26 (*n* = 49)	3.61 (*n* = 7)	1.03 (*n* = 2)	0.52 (*n* = 1)	0.00	0.52 (*n* = 1)	0.00
40–44	43.02 (*n* = 84)	11.27 (*n* = 22)	3.58 (*n* = 7)	1.02 (*n* = 2)	0.00	0.00	0.51 (*n* = 1)
45–49	39.06 (*n* = 80)	16.60 (*n* = 34)	7.32 (*n* = 15)	0.49 (*n* = 1)	0.00	0.49 (*n* = 1)	0.00
50–54	89.29 (*n* = 117)	23.66 (*n* = 31)	26.71 (*n* = 35)	2.29 (*n* = 3)	2.29 (*n* = 3)	1.53 (*n* = 2)	0.00
55–59	73.13 (*n* = 123)	30.92 (*n* = 52)	38.65 (*n* = 65)	1.78 (*n* = 3)	0.00	2.38 (*n* = 4)	0.00
60–64	68.25 (*n* = 118)	35.86 (*n* = 62)	50.90 (*n* = 88)	4.05 (*n* = 7)	0.00	1.16 (*n* = 2)	0.00
65–69	47.17 (*n* = 68)	31.21 (*n* = 45)	47.17 (*n* = 68)	7.63 (*n* = 11)	0.00	2.08 (*n* = 3)	0.00
70–74	60.72 (*n* = 57)	52.20 (*n* = 49)	47.94 (*n* = 45)	14.91 (*n* = 14)	2.13 (*n* = 2)	2.13 (*n* = 2)	0.00
75–79	43.30 (*n* = 44)	45.27 (*n* = 46)	32.48 (*n* = 33)	16.73 (*n* = 17)	3.94 (*n* = 4)	0.00	0.00
80–84	27.44 (*n* = 18)	47.26 (*n* = 31)	33.54 (*n* = 22)	12.20 (*n* = 8)	7.62 (*n* = 5)	0.00	0.00
85+	26.71 (*n* = 13)	26.71 (*n* = 13)	10.27 (*n* = 5)	2.05 (*n* = 1)	4.11 (*n* = 2)	0.00	0.00
Incidence density rate	32.92 (*n* = 803)	16.56 (*n* = 404)	15.91 (*n* = 388)	2.79 (*n* = 68)	0.66 (*n* = 16)	0.61 (*n* = 15)	0.04 (*n* = 1)

Note: *n*: the number of women in each age category.

**Table 2:** Age-related Relative Risk for genital cancer.

Cancer Types	Cases <50 Years Old	Cases >50 Years Old	RR for Cancer >50 Years Old vs. <50 Years Old	95% CI	*p* Value
Vaginal cancer	0	16	38.5129	2.3105 to 641.9584	<0.0110
Vulvar cancer	4	64	18.6762	6.8014 to 51.2838	<0.0001
Endometrial cancer	27	361	15.5001	10.4844 to 22.9152	<0.0001
Uterine sarcoma	2	13	7.585	1.7117 to 33.6115	<0.007
Ovarian cancer	75	329	5.1041	3.9726 to 6.558	<0.0001
Cervical cancer	245	558	2.6305	2.2641 to 3.056	<0.0001

Note: RR: Relative Risk. CI: Confidence interval.

**Table 3:** Stages in cases with genital cancer.

Cancer Type *p* < 0.0001	In Situ Stage 0	Stage I	Stage II	Stage III	Stage IV	Total Patients	
						No.	%
Cervical	69	13	99	260	362	803	47.37
Endometrial	133	2	42	86	125	388	22.89
Ovarian	46	3	20	52	283	404	23.83
Placenta	0	0	0	0	1	1	0.058
Uterine sarcoma	0	0	0	10	5	15	0.88
Vaginal	1	0	0	4	11	16	0.943
Vulvar	3	0	6	20	39	68	4.011
Total	252	18	167	432	826	1695	100

**Table 4:** PYLL in women aged 20–85 and over by cancer type, Arad, Romania.

Cause of Premature Death	ICD-10	ICD-11 [19]	Person-Years Lost	Cases	Deaths	PYLL Per Death	Percent %	Death Rate
Cervical cancer	C53	2C77.Z	13,927	803	438	17.34	47.37	54.55
Ovarian cancer	C56	2C73.Z	4860	404	277	12.03	23.83	68.56
Endometrial cancer	C54-C55	2C76.Z	3081	388	142	7.94	22.89	36.60
Vulvar cancer	C51	2C70.Z	493	68	42	7.25	4.01	61.76
Uterine sarcoma	C54	2B5F.0	255	16	10	15.94	0.94	62.50
Vaginal cancer	C52	2C71.Z	56	15	8	3.73	0.88	53.33
Placenta cancer	C58	2C75.Z	36	1	1	36.00	0.06	100.00
Total	-	-	22,708	1695	918	13.40	100	54.16

ICD—International Classification of Diseases; PYLL—Years of potential life lost.

**Table 5:** YLL, YLD, and DALY in women aged 20–85 and over by genital cancer, Arad, Romania.

**Index**	**Item**	**Deaths**	**Deaths per 1000**	**Av. Age at Death**	**Standard** **LE**	**YLLs**	**YLL per 1000**
YLL	Genital cancers	918	0.0003	60.9	78.5	14,294.8	58.6
YLL	Cervical cancers	421	0.0001	56.8	78.5	7703	31.6
YLL	Study age groups Genital cancers	918	3.76	60.87	78.5	14,294.8	58.6
YLL	Study age groups Cervical cancers	421	1.7	56.8	78.5	7703	31.6
**YLD**	**Incidence**	**Incidence** **per 1000**	**Age** **at Onset**	**Duration** **(years)**	**Disability Weight**	**YLDs**	**YLD per 1000**
Genital cancers	1695	6.9	60.2	2.6	0.5	2103.4	8.6
Cervical cancers	803	3.3	56.2	4.7	4.4	350	1.4
**DALY**		**DALYs**		**DALYs per 1000**			
Genital cancers		16,397.2		67.2			
Cervical cancers		8053		33.0			

LE—Life Expectancy, YLL—Years of Life Lost due to premature mortality; YLD—Years Lived with Disability; DALY—Disability-Adjusted Life Year; DALY = YLL + YLD.

## Appendix A

**Table A1:** In situ histopathological staging.

Cancer Type	Histology	In Situ No. of Cases
Cervical cancer	Adenocarcinoma in situ	31
	Carcinoma in situ	9
	Squamous-cell carcinoma in situ	29
Endometrial cancer	Adenocarcinoma in situ	131
	Adenosquamous carcinoma in situ	1
	Squamous-cell carcinoma in situ	1
Ovarian cancer	Ovarian carcinoma in situ	46
Vaginal cancer	Adenocarcinoma in situ	1
Vulvar cancer	Adenocarcinoma in situ	1
	Carcinoma in situ	1
	Squamous-cell carcinoma in situ	1
Total		252

**Table A2:** Histopathological staging for all female genital cancers.

Genital Cancers		Stages				
		0	I	II	III	IV
Adenocarcinoma in situ		31	0	0	0	0
Carcinoma in situ		9	0	0	0	0
Clear cell adenocarcinoma		0	0	0	2	1
Endometrioid adenocarcinoma		0	0	6	4	6
Mucinous adenocarcinoma		0	0	0	1	5
Papillary carcinoma		0	0	2	0	1
Primary cervical malignant melanoma		0	0	0	1	0
Serous papillary adenocarcinoma		0	0	0	2	1
Small cell carcinoma		0	0	0	2	0
Squamous-cell carcinoma		0	13	91	248	347
Squamous-cell carcinoma in situ		29	0	0	0	0
Verrucous carcinoma		0	0	0	0	1
Endometrial cancer	Adenocarcinoma in situ	131	0	0	0	0
	Adeno-squamous carcinoma	0	0	0	8	4
	Adenosquamous carcinoma in situ	1	0	0	0	0
	Clear cell carcinoma	0	0	1	4	2
	Endometrioid adenocarcinoma	0	1	27	48	53
	Mixed carcinoma	0	1	2	6	43
	Mucinous carcinoma	0	0	0	1	0
	Papillary carcinoma	0	0	4	10	5
	Serous papillary carcinoma	0	0	3	1	2
	Squamous-cell carcinoma	0	0	3	5	4
	Squamous-cell carcinoma in situ	1	0	0	0	0
	Undifferentiated carcinoma	0	0	2	3	12
Ovarian cancer	Clear cell adenocarcinoma	0	0	2	5	15
	Endometrioid adenocarcinoma	0	0	5	7	46
	Metastatic adenocarcinoma to the ovary	0	0	0	0	4
	Mucinous adenocarcinoma	0	1	3	11	70
	Ovarian carcinoma in situ	46	0	0	0	0
	Ovarian choriocarcinoma	0	0	0	0	1
	Papillary carcinoma	0	1	1	1	7
	Serous adenocarcinoma	0	1	2	21	107
	Serous carcinoma	0	0	7	7	33
Placenta cancer	Gestational trophoblastic tumor	0	0	0	0	1
Uterine sarcoma	Fibrosarcoma of the uterus	0	0	0	2	3
	Leiomyosarcoma	0	0	0	1	0
	Rhabdomyosarcoma of the uterus	0	0	0	6	1
	Stromal sarcoma	0	0	0	1	1
Vaginal cancer	Adenocarcinoma in situ	1	0	0	0	0
	Malignant melanoma	0	0	0	3	3
	Squamous epithelial carcinoma	0	0	0	1	5
	Squamous-cell carcinoma	0	0	0	0	3
Vulvar cancer	Adenocarcinoma in situ	1	0	0	0	0
	Basal-cell carcinoma	0	0	0	2	4
	Carcinoma in situ	1	0	0	0	0
	Malignant melanoma	0	0	2	1	8
	Papillary carcinoma	0	0	0	0	1
	Squamous-cell carcinoma	0	0	4	17	26
	Squamous-cell carcinoma in situ	1	0	0	0	0
Total		252	18	167	432	826
